# Rationale and Design of a Multi-National Study of Physicians’ Opinions, Attitudes, and Practices Regarding Influenza Vaccination in Patients with Cardiovascular Diseases: A Mixed Methods Designs. The FLUence Project

**DOI:** 10.5334/gh.1358

**Published:** 2024-10-15

**Authors:** Sebastián Garcia-Zamora, Angela S. Koh, Svetlana Stoica, Nariman Sepehrvand, Harish Ranjani, Salisu Ishaku, Naomi Herz, Vanessa Kandoole-Kabwere, Pablo Perel, Amitava Banerjee, Charlotte Warren-Gash, Sean Taylor, Daniel José Piñeiro, María Inés Sosa-Liprandi, álvaro Sosa-Liprandi

**Affiliations:** 1 Department of Research Methodology and Evidence-Based Medicine, Faculty of Medicine, National University of Rosario (UNR), Argentina; 2 Cardiology Department, Delta Clinic, Rosario, Argentina; 3 Emerging Leaders Programme, Cohort 2022, World Heart Federation, Switzerland; 4 Department of Cardiology, National Heart Centre Singapore, 5 Hospital Drive, 169609, Singapore; 5 Duke-NUS Medical School, 8 College Road, 169857, Singapore; 6 Institute for Cardiovascular Diseases Timisoara, Romania; 7 ‘Victor Babes’ University of Medicine and Pharmacy Timisoara, Romania; 8 Canadian VIGOUR Centre, and Department of Medicine, University of Alberta, Edmonton, Alberta, Canada; 9 Department of Medicine, University of Calgary, Calgary, Alberta, Canada; 10 Madras Diabetes Research Foundation & Dr. Mohan’s Diabetes Specialities Centre, Chennai & Bengaluru, India; 11 Equity in Health and Research Initiative Nigeria AND Julius Global Health, University Medical Center, Utrecht, the Netherlands; 12 British Heart Foundation, United Kingdom; 13 Malawi Liverpool Wellcome Trust, United Kingdom; 14 Department of Non-Communicable Disease Epidemiology, Faculty of Epidemiology and Population Health, London School of Hygiene & Tropical Medicine, London, United Kingdom; 15 World Heart Federation, Geneva, Switzerland; 16 Department of Cardiology, Barts Health NHS Trust, London, United Kingdom; 17 Institute of Health Informatics, University College London, London, United Kingdom; 18 Department of Medicine, University of Buenos Aires, Buenos Aires, Argentina; 19 Cardiology Department, Sanatorio Güemes, Ciudad Autónoma de Buenos Aires, Argentina

**Keywords:** influenza, vaccine, inflammation, cardiovascular disease, survey

## Abstract

Infections, particularly those involving the respiratory tract, are associated with an increased incidence of cardiovascular events, both de novo and as exacerbations of pre-existing cardiovascular diseases. Influenza vaccination has consistently been shown to reduce the incidence of cardiovascular events. Nonetheless, vaccination rates among adults remain suboptimal, both in the general population and among high-risk individuals. Multiple barriers hinder achieving adequate vaccination rates, with physicians’ beliefs and attitudes towards these interventions being crucial.

The FLUence project was developed within the framework of the World Heart Federation’s Emerging Leaders program, to address this issue. This project has two phases: a global quantitative survey to assess the perceptions, opinions, and attitudes and challenges of physicians worldwide regarding the safety and efficacy of the influenza vaccination use, and a qualitative survey to further investigate the barriers and facilitators of recommending and using this vaccination. The quantitative survey was created and disseminated in five languages (English, Spanish, French, Italian, and Portuguese) to physicians of all specialties who care for adults, with a particular focus on patients with cardiovascular disease. The survey included eight domains with a total of 36 questions with closed options; a Likert scale with five possible answers was used to gauge participants’ opinions.

To gain deeper insights into the complexities behind the low vaccination rates in adults, the second part of the project comprises a qualitative survey, conducted in the two lower-middle- and upper-middle-income countries: India and Argentina, respectively. These countries were selected because patients with cardiovascular diseases have access to free influenza vaccination in Argentina, whereas patients must pay for the vaccine out of pocket in India.

Thus, the FLUence study will provide valuable information to better understand the perceptions and barriers to improving influenza vaccination rates from the perspective of physicians. It is imperative to actively engage all healthcare providers to improve influenza vaccination rates.

## Background

For centuries, infectious diseases were the leading cause of death worldwide. From the early twentieth century, changes in the environment, by means of urbanization, improved sanitation and the advent of simple preventive and therapeutic measures such as vaccination and antibiotics, led to a reduction in the burden of infectious diseases (1,2,3). Cardiovascular diseases (CVD) have subsequently become the leading cause of death worldwide with an estimated 17.9 million people dying from CVD in 2019 (4). It is worth noting that in Low- and Middle-Income Countries (LMICs), while infectious diseases continue to show a significant burden, CVD and other non-communicable diseases have now taken over (5).

Over the past few decades, there is increasing evidence to suggest links between acute infections (both viral and bacterial) and cardiovascular events (6,7,8,9). In several observational studies, acute thrombotic events such as acute coronary syndrome (ACS) and stroke were seen to be associated with the occurrence of a preceding acute infection, in particular infections of the respiratory tract (1,6,7,8,9,10,11,12,13,14). Up to one-third of ACS events are preceded by respiratory symptoms (6,15,16). This risk is highest during the first days after infection and gradually decreases over time (10,13,15,17,18). In addition, respiratory infections have been associated with a worsening of pre-existing cardiovascular conditions (1,19,20,21,22).

A systematic review of observational studies demonstrated that influenza is a trigger for acute myocardial infarction with a seasonal effect observed such that increased mortality due to cardiovascular disease were reported during times when influenza was circulating (11). In this context, several systematic reviews and meta-analyses have shown that influenza vaccines reduce cardiovascular morbimortality (23,24,25,26). Furthermore, the IAMI trial randomized patients after an acute myocardial infarction or high-risk percutaneous angioplasty and found that those who received the influenza vaccine during the hospitalization exhibited fewer major adverse cardiovascular events at 12 months of follow-up (27).

This comprehensive evidence has led scientific societies to develop consensus guidelines regarding influenza vaccination for high-risk patients with CVD (28,29,30,31). However, available data suggest that in most countries, vaccination rates fall far below the World Heart Organization’s 75% target for high-risk patients (32,33,34,35). For instance, data from the general population in Argentina indicate a vaccination rate of 37.7% (32), while the rate among patients with cardiovascular disease is about 65% (33). In India, studies have shown that influenza vaccination rates among physicians range from 4.4% to 54.8% (36). In a community survey conducted in Pune, India, only 8.3% of participants reported having received the influenza vaccine, and 10.6% stated that a household member had received it (37).

Several factors, including patient, provider, and access issues are implicated in the low uptake of influenza vaccination in various settings (38). There is a paucity of data regarding what factors on the provider-side contribute to the low uptake in patients with CVD. Hence, in the FLUence project, we aim to explore the physician’s knowledge, attitude, and practice regarding influenza vaccination among patients with cardiovascular diseases and to investigate the variability of these factors across different settings.

## Methods and design

### Study design

The FLUence project will employ a mixed-methods approach, incorporating both a quantitative survey and a qualitative component, which will include in-depth interviews conducted in India and Argentina.

#### Quantitative survey

A cross-sectional electronic survey was designed and implemented following the Consensus-Based Checklist for Reporting of Survey Studies (CROSS) proposed by the Enhancing the Quality and Transparency of Health Research (EQUATOR) Network and the CHERRIES checklist (39,40). The survey was conducted from October 2023 to June 2024. Using *SurveyMonkey^®^* we prevented duplicate responses from the same IP addresses.

The survey included eight domains with a total of 36 questions with closed options. The selected domains were:

- General information
- Knowledge
- Perceived risk
- Perceived benefits
- Self-efficacy
- Knowledge gaps and training needs
- Barriers to immunization
- Final questions related to practical implementation

These questions aimed to capture demographics, profession, work environment, level of work experience, and the knowledge, attitude, and practice of participants regarding prescribing or encouraging influenza vaccination among patients with CVD. It also captured the current practice processes in the participants’ jurisdictions in terms of vaccine prescribers/providers and inclusion of those recommendations in regional and local professional guidelines (Supplementary material I).

A Likert scale with five possible answers was used to gauge participants’ opinions on different aspects of vaccination. The only exception was the question related to respondents’ opinion regarding the benefit of influenza vaccine to patients with high cardiovascular risk, for which we provided four options: ‘No benefit,’ ‘Little benefit,’ ‘Great benefit,’ and ‘Not sure.’

The questionnaire link was distributed via email through the World Heart Federation mailing list and other associated scientific societies, in five languages: English, Spanish, French, Italian, and Portuguese.

##### Study population

Physicians from any country who provide care to adult patients were invited to participate in the quantitative survey. We aim to reach over 3,500 physicians worldwide.

Participation in the survey is voluntary, and all respondents have the option to decline participation. To protect and ensure the confidentiality of participants’ personal information, informed consent was implied by voluntary completion of the survey (*see Ethical consideration*).

#### Qualitative survey

The qualitative phase of the study will be conducted between August 2024 and March 2025. We will utilize a qualitative method of inquiry through in-depth, semi-structured interviews with stakeholders involved in the delivery of CVD prevention and management. These interviews will gather rich data grounded in personal experiences, considering the interviewees as experts in their own experiences. This approach will help identify the barriers and facilitators to the uptake of influenza vaccination in patients with cardiovascular diseases in the two lower-middle- and upper-middle-income countries: India and Argentina, respectively. Notably, the influenza vaccine is freely available for people with cardiovascular diseases in Argentina.

The qualitative interviews will collect data on providers’ thoughts, feelings, motivations, and the social processes and practical issues that drive or hinder vaccination. This information will help develop evidence-informed strategies to increase uptake among high-risk patients and the general public.

**Figure 1:**
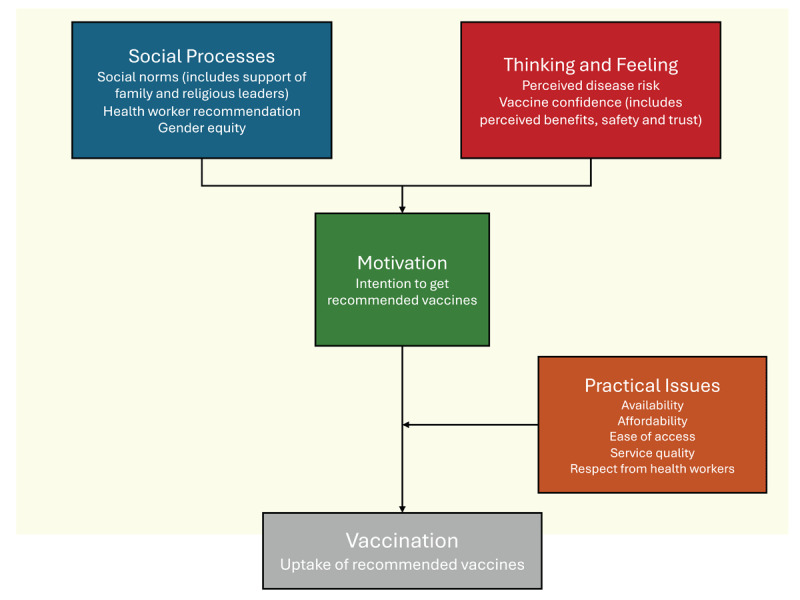
The Behavioural and Social Drivers (BeSD) framework. Adapted with modifications from reference (41): ‘Behavioural and social drivers of vaccination: Tools and practical guidance for achieving high uptake’, World Health Organization 2022.

We have employed the Behavioural and Social Drivers (BeSD) framework (Figure 1) for the qualitative phase of the study (41). This framework enables vaccination uptake programs to design, target, and evaluate interventions for greater impact and efficiency and to examine and understand trends over time. Briefly, the behavioral and social drivers of vaccination are defined as beliefs and experiences specific to vaccination that are potentially modifiable to increase vaccine uptake (41). Typically, four domains will be measured by this framework (Figure 1):

- thoughts and feelings about vaccines.
- social processes that drive or inhibit vaccination.
- motivation or hesitancy to seek vaccination.
- practical issues involved in seeking and receiving vaccination.

Specific to this qualitative section of our proposal, the BeSD framework allows the capture of broader factors influencing stakeholders’ responses, such as political perspectives, socio-economic status, literacy, or other factors not covered in the quantitative survey.

##### Study setting and recruitment

**Figure 2:**
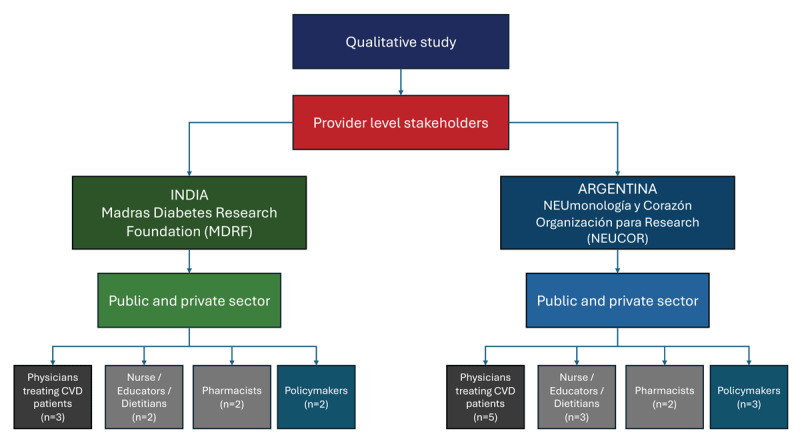
Recruitment of the participants for the qualitative phase of the project.

We will recruit provider-level stakeholders identified by the researchers. Recruitment of stakeholders will be conducted in both public and private healthcare settings in India and Argentina. In each of these countries, we will include physicians treating patients with CVD (n = 8), nurses, educators, dietitians (n = 5), pharmacists (n = 4), and policymakers (n = 5). The two partner institutions for recruitment and conducting the qualitative interviews are the Madras Diabetes Research Foundation (MDRF) in India and NEUmonologia y Corazon Organization for Research (NEUCOR) in Argentina (see Figure 2, *Flow Chart Diagram*). Recruitment for healthcare providers and policymakers will occur through the extensive local networks of the research team.

##### Interview guide development and interview process

The interview will be based on the Behavioral and Social Drivers (BeSD) framework, supplemented by insights from other relevant previous studies (41,42). This approach ensures that the guide is comprehensive and tailored specifically to the health provider stakeholder group, as detailed in Table 1. Interviews will be conducted by a trained qualitative researcher who is fluent in the participants’ language, ensuring clear and effective communication. Each interview will be scheduled to last approximately one hour, providing ample time to explore the topics in depth. The interviews will be conducted in a private setting to ensure confidentiality and encourage open and honest responses from participants.

**Table 1:** Conceptual framework for the qualitative study.


BARRIERS AND FACILITATORS IN THE UPTAKE OF INFLUENZA VACCINATION IN PATIENTS WITH CARDIOVASCULAR DISEASES IN INDIA AND ARGENTINA	

CATEGORY	FACILITATORS AND BARRIERS

Health Care provider/Physicians	Awareness, need, misinformation, motivation, self-belief, patient hesitation

Health care provider/other groups	Awareness, self-belief, other sources, negative perception, patient hesitation

Policy makers	Insurance coverage, bureaucracy hurdles


Each interview will be audio-recorded with the participants’ consent, allowing for detailed and accurate data collection. The recordings will facilitate thorough review and analysis, ensuring that nuanced insights and key points are captured. To enhance the reliability of the data, the interviewer will immediately transcribe their initial reflections and observations following each interview. These transcriptions will include notes on non-verbal cues, the interview context, and any emergent themes or unexpected topics discussed during the session.

##### Ethical consideration

The project has been approved by the Executive Board of the World Heart Federation and is currently under review by the Ethics Committee of the Madras Diabetes Research Foundation in India and the Ethics Committee of the Faculty of Medicine at the National University of Rosario (UNR) in Argentina. To ensure the honesty of responses without fear of judgment, and given that all respondents were healthcare providers, participants were not asked to complete a written informed consent form. Instead, consent was implied by the voluntary completion of the survey.

##### Data analysis of the quantitative survey

A non-probability snowball sampling will be performed. Demographics and participant characteristics will be presented using descriptive statistics. Continuous variables will be expressed as mean and standard deviation or median and interquartile range, according to their distribution. The normality of each variable will be evaluated using graphic tools (histograms and normal probability plots) and the Shapiro-Wilk test. The categorical variables will be presented as numbers and percentages.

We will categorize the participants based on the scores in terms of knowledge, attitude, and practice and will compare the levels across the participating sites using the Chi-square test or Fisher’s exact test depending on the frequency of expected values. The student’s T-test will be used for comparisons of the normally distributed continuous variables between groups.

Multiple logistic regression models will be constructed to explore the variables associated with positive attitudes and practices toward influenza vaccination. All variables that achieve a p-value of ≤0.2 in the univariate model will be included in the multivariable regression model, along with those deemed clinically relevant by the investigators. All tests will be two-tailed, and a p-value <0.05 will be considered statistically significant. The analyses will be performed using STATA (Version 18.0, Stata Corp., College Station, TX, USA).

##### Qualitative analysis

Interviews will be transcribed by trained researchers and uploaded into QSR Nvivo and ATLAS.ti, specialized software for data management and analysis. We will employ a combination of deductive and inductive coding methods: deductive coding based on the conceptual framework and inductive coding derived from emerging themes in the data. A rigorous thematic analysis approach will be applied to ensure an accurate representation of the collected data.

In each country (Argentina and India), two experienced qualitative researchers will familiarize themselves with the data by listening to recordings and reading interview transcripts. Subsequently, they will independently apply descriptive codes to short segments of the interview data, both manually and within QSR Nvivo and ATLAS.ti. Codes will then be organized into overarching themes, with careful consideration of divergent data and illustrative quotes. The researchers will compare, contrast, and integrate their findings. Results will be presented to the entire research team for consensus and refinement of themes. Other researchers interested in reviewing the raw data (interview transcripts) will be encouraged to do so. The reporting of qualitative data in academic journals will adhere to the COREQ checklist (43).

## Discussion

Low vaccination rates among adults are widespread issues globally (1,28,29,32,33,38,41). Although numerous factors contribute to this problem, research consistently shows that healthcare professionals are key influencers in vaccination uptake (38,41). Studies have demonstrated that when physicians proactively recommend vaccinations, immunization rates rise significantly (38). To improve vaccination rates, it is crucial to understand the fears and barriers that prevent physicians from actively promoting vaccines in the general adult population or certain high-risk patients such as those with CVD. These barriers may include concerns about vaccine safety, lack of time during patient consultations, insufficient knowledge about the latest vaccine recommendations, or perceived patient resistance (38,41,44). Addressing these concerns through targeted education and support can empower physicians to become more active advocates for vaccination (1,45).

Preventing cardiovascular events is a cornerstone, with advancements in this area significantly improving life expectancy and quality of life. Vaccines are an essential tool for reducing residual cardiovascular risk, making their promotion a critical task for healthcare providers (1,46). The challenge lies in developing and implementing strategies that ensure high vaccination rates, thereby transforming healthcare professionals into facilitators of immunization rather than barriers (1,38).

Thus, our study will provide critical contextual data on the barriers and facilitators to influenza vaccination uptake in patients with cardiovascular diseases across multiple countries around the world from a healthcare practitioner point of view (Central Illustration). The results will help identify current gaps, inform key recommendations to practitioners, policy makers, and researchers on how to create and deliver vaccination uptake programs to achieve greater uptake rates. Furthermore, most of the available literature dates from the pre-COVID-19 era. During the COVID-19 pandemic, most countries have not achieved high influenza vaccine uptake rates (32,33,47,48); even in high uptake settings such as England, uptake remains suboptimal among younger people with cardiovascular disease (49).

**Central Illustration:**
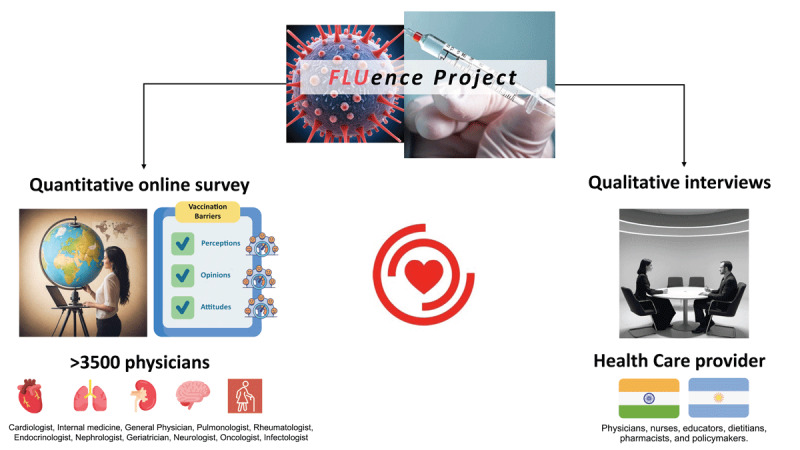
Schematic diagram of the design and phases of the FLUence project. Icons were adapted from Freepik, turkkub, Khoirul Huda, Futuer, Iconpro86, Paul J, Ifanicon, and kmg design from www.flaticon.com.

### Anticipated Limitations

The FLUence project will have some limitations that deserve to be highlighted. First, the survey is restricted to physicians; this narrow focus means that the survey will not capture barriers and challenges faced by other stakeholders, such as patients, healthcare administrators, or logistical and access issues. While this concentrated approach allows for more detailed and relevant questions for physicians, it limits the overall understanding of the multifaceted barriers to influenza vaccination. Second, the survey will target a broad spectrum of physicians from various medical specialties. While this diverse participation can provide a comprehensive view of vaccination practices globally, it may also introduce significant heterogeneity. Different geographical and professional contexts might necessitate tailored intervention strategies, which could complicate the implementation of uniform policies based on the survey results.

Finally, the qualitative phase of the project will be conducted only in two lower-middle- and upper-middle-income countries, India and Argentina. While these insights will be valuable for understanding the local barriers and facilitators of influenza vaccination, they may not be entirely generalizable to other regions, especially to those of practitioners in higher-income countries.

## Conclusion

There are multiple determinants contributing to low influenza vaccination rates, even among high-risk adults. In this context, the FLUence project will provide invaluable insights by offering both quantitative and qualitative perspectives from physicians across various specialties. This approach will assess their perceptions and opinions on the implications of this issue, thus identifying potential strategies for enhancement.

Hence, it is imperative that all healthcare professionals adopt a proactive and supportive approach towards adult immunizations to enhance global and cardiovascular health outcomes for all individuals.

## Additional File

The additional file for this article can be found as follows:

10.5334/gh.1358.s1 Supplementary Materials. Survey Used During The Quantitative Phase of The Study.
