# Supplementary material for: Rationale and Design of a Multi-National Study of Physicians’ Opinions, Attitudes, and Practices Regarding Influenza Vaccination in Patients with Cardiovascular Diseases: A Mixed Methods Designs. The FLUence Project
Source: Glob Heart. 2024 Oct 15;19(1):78. doi: 10.5334/gh.1358 (PMC11488190; doi:10.5334/gh.1358)
Supplement: Supplementary Materials. — Survey Used During The Quantitative Phase of The Study. [file gh-19-1-1358-s1.pdf]

SUPPLEMENTARY MATERIALS

Rationale and design of a multi-national study of physicians' opinions, attitudes, and practices regarding Influenza vaccination in patients with cardiovascular diseases: a mixed methods designs. The FLUence project.

Sebastián Garcia-Zamora[1,2,3], Angela S. Koh[3,4,5], Svetlana Stoica[3,6,7], Nariman Sepehrvand[3,8,9], Harish Ranjani[3,10], Salisu Ishaku[3,11], Naomi Herz[3,12], Vanessa Kandoole-Kabwere[3,13], Pablo Perel[14,15], Amitava Banerjee[15,16,17], Charlotte Warren-Gash[14], Sean Taylor[15], Daniel J. Piñeiro[18], María Inés Sosa-Liprandi[19], Álvaro Sosa-Liprandi[19].

1- Department of Research Methodology and Evidence-Based Medicine, Faculty of Medicine, National University of Rosario (UNR), Argentina.

2- Cardiology Department, Delta Clinic, Rosario, Argentina.

3- Emerging Leaders Programme, Cohort 2022, World Heart Federation.

4- Department of Cardiology, National Heart Centre Singapore, 5 Hospital Drive, 169609, Singapore.

5- Duke-NUS Medical School, 8 College Road, 169857, Singapore.

6- Institute for Cardiovascular Diseases Timisoara, Romania.

7- "Victor Babes" University of Medicine and Pharmacy Timisoara, Romania.

8- Canadian VIGOUR Centre, and Department of Medicine, University of Alberta, Edmonton, Alberta, Canada.

9- Department of Medicine, University of Calgary, Calgary, Alberta, Canada

10- Madras Diabetes Research Foundation & Dr. Mohan's Diabetes Specilaities Centre, Chennai & Bengaluru, India.

11- Equity in Health and Research Initiative Nigeria AND Julius Global Health, University Medical Center, Utrecht, the Netherlands.

12- British Heart Foundation, United Kingdom.

13- Malawi Liverpool Wellcome Trust, United Kingdom.

14- Department of Non-Communicable Disease Epidemiology, Faculty of Epidemiology and Population Health, London School of Hygiene & Tropical Medicine, London, United Kingdom.

15- World Heart Federation, Geneva, Switzerland.

16- Department of Cardiology, Barts Health NHS Trust, London, United Kingdom.

17- Institute of Health Informatics, University College London, London, United Kingdom.
18- Department of Medicine, University of Buenos Aires, Buenos Aires, Argentina.
19- Cardiology Department, Sanatorio Güemes, Ciudad Autónoma de Buenos Aires, Argentina.

SURVEY USED DURING THE QUANTITATIVE PHASE OF THE STUDY

Brief description of the survey:

Dear colleague, the purpose of this survey is to understand your opinion about influenza vaccination in adult patients, with a focus on people living with cardiovascular diseases. This survey is voluntary, and all of your responses, as well as your participation in the survey itself, will be anonymized. Participating in this project will take less than 4 minutes, and your contribution will greatly assist us.

By answering the survey, you are providing your consent to be part of this project.

Thank you very much for your help!

Questions:

General information:

- Country [list of options]
- Age
- Sex
- Specialty
    * Cardiologist
    * Internal medicine
    * General Physician
    * Pulmonologist
    * Rheumatologist
    * Endocrinologist
    * Nephrologist
    * Geriatrician
    * Neurologist
    * Oncologist
    * Critical care / Emergency
    * Infectologist
    * Others
- Are you currently in training? Yes - No
- How long have you been practicing in your specialization (in years)?
- Health Care Setting
    i) Public hospital

ii) Private hospital

iii) Public health clinic/ primary care

iv) Private health clinic/ primary care

- Have you been vaccinated against influenza in the last 12 months? No - Yes - I don't remember

<u>Knowledge:</u>

- Do you know if the influenza vaccine is available in your country? No - Yes - Not sure
- Do you know if the influenza vaccine is covered by social insurance, government or any other health insurance in your country? No - Yes - Not sure
- Do you know if high risk cardiovascular patients need to pay anything to get the influenza vaccine in your country? No - Yes - Not sure
- In your country, what providers are responsible for prescribing influenza vaccination? (Select all that apply):

    * Physicians
    * Pharmacists
    * Nurses
    * Nurse practitioners
    * Others
    * None

- Have you ever prescribed the influenza vaccine to any patient? No - Yes - Not sure
- Do you know if there is an age in your country after which all adults should be vaccinated, regardless of their comorbidities? No - Not sure - Yes (if yes, please specify)
- Do you know if the influenza vaccine is considered an essential medicine in your country? No - Yes - Not sure
- Do you know of any general or specific guidelines that make recommendations about influenza vaccination in patients with cardiovascular disease?

    * No
    * Not sure
    * Yes, national guidelines
    * Yes, international guidelines
    * Yes, national and international guidelines

- In your country, do you know whether there are different types of influenza vaccine for adults, according to their age or comorbidities? No - Yes - Not sure

Perceived Risk/ Susceptibility

- On a scale from 1 to 10, how safe do you believe the influenza vaccine is in the adult general population? (1 being the most dangerous and 10 the most safer one)
- On a scale from 1 to 10, how safe do you believe the influenza vaccine is in patients under antiplatelet or anticoagulation treatment? (1 being the most dangerous and 10 the most safer one)
- On a scale from 1 to 10, how serious would it be if your high risk cardiovascular patients contract influenza? (1 being not very serious and 10 the most serious disease)
- If a patient has an acute coronary syndrome, how much time do you think should elapse before safely prescribing the influenza vaccine?
    * Not sure
    * At least 6 months after being discharged
    * Between 3 to 6 months after being discharged
    * Between 1 to 3 months after being discharged
    * During hospitalization or during the 1st month after being discharged
- In a patient with heart failure that has been hospitalized, how much time do you think should elapse since hospitalization to safely prescribe the influenza vaccine?
    * Not sure
    * At least 6 months after being discharged
    * Between 3 to 6 months after being discharged
    * Between 1 to 3 months after being discharged
    * Could be vaccinated during hospitalization or soon after being discharged
- On a scale from 1 to 10, how often do you think adverse effects of any kind occur after influenza vaccination? (1 being very infrequently and 10 very frequent)
- On a scale from 1 to 10, how often do you think **serious** adverse events occur after influenza vaccination?? (1 being very infrequently and 10 very frequent)

Note: serious adverse events as defined by WHO: It causes the death of the vaccinated individual; or It puts the life of the vaccinated individual in imminent danger; or leads to hospitalization or prolongation of hospital stay; or causes persistent or significant disability or incapacity.

Perceived Benefits

- On a scale from 1 to 5, how important do you believe the influenza vaccine is for patients aged 65 years and above irrespective of any pre-existing comorbidities? (1 being the least important intervention and 5 the most important intervention)

- On a scale from 1 to 5, how important do you believe the influenza vaccine is for older patients with cardiovascular disease aged 65 years and above? (especially acute or chronic coronary syndrome or heart failure)? (1 being the least important intervention and 5 the most important intervention)

- On a scale from 1 to 5, how important do you believe the influenza vaccine is for younger patients with cardiovascular disease aged below 65 years old? (especially acute or chronic coronary syndrome or heart failure)? (1 being the least important intervention and 5 the most important intervention)

- How likely do you think that the influenza vaccine could provide some benefit to a patient with high cardiovascular risk?

(Put an X where you consider the most suitable probability of benefit or not)

	No confidence	Little Confidence	Very confidence	Not sure
Prevent getting the flu				
Reduce myocardial infarction risk				
Reduce stroke risk				
Reduce mortality risk				
Reduce hospitalization for any reason				

<u>Self-Efficacy</u>

- On a scale of 1 to 10, how comfortable are you with talking to patients about the influenza vaccine risk and benefits? (1 being not comfortable at all and 10 being very comfortable)

- On a scale of 1 to 10, do you feel you need training to discuss the risks and benefits of the influenza vaccine with patients? (1 being “I don’t need training” and 10 “I definitely need training”)

<u>Information needs</u>

- On a scale from 1 to 10, how important do you think it is to receive information about influenza vaccination for your daily practice? (1 being the not at all useful and 10 being extremely useful)

- What tools/job aids do you need to effectively increase influenza vaccine discussion among your patients? Select all that apply:

i) Clinical time/ reduction of clinical overload/ allied health care worker support

ii) Educational resources

iii) Monetary benefits for your patients (for example, subsidized care for influenza among patients who contract influenza despite being receiving influenza vaccination in last 12 months)

iv) Information for your patients about influenza vaccine

v) None of the above

<u>Barriers</u>

- In your country, do you believe that any of the following factors affect access to the influenza vaccine? (Put an X where you consider the most suitable)

	Major barrier	Minor barrier	Could be a barrier	It's not a barrier	I don't know
Cost of the influenza vaccine					
Availability of the influenza vaccine					
Patients' beliefs about influenza vaccine					
Belief of the Physician about risk-benefits of influenza vaccine					
Physicians' fear of side effects of influenza vaccine					
Patients' fears of side effects of influenza vaccine					

<u>Final questions</u>

- Do you have any checklist or other kind of reminder to prescribe high risk patients the influenza vaccine? No - Yes - Not sure.

- Have you ever attended courses, brief meetings, or classes on the relationship between influenza infection and its cardiovascular effects? No - Yes - I don't remember.

- On a scale from 1 to 10, how relevant do you consider this topic is in your daily practice?
(1 being not at all relevant and 10 being very relevant)
